# Influence of hypoxic stimulation on angiogenesis and satellite cells in mouse skeletal muscle

**DOI:** 10.1371/journal.pone.0207040

**Published:** 2018-11-08

**Authors:** Hiroshi Nagahisa, Hirofumi Miyata

**Affiliations:** Biological Sciences, Graduate School of Sciences and Technology for Innovation, Yamaguchi University, Yamaguchi, Japan; University of Minnesota Medical Center, UNITED STATES

## Abstract

We clarified in our previous study that hypoxic training promotes angiogenesis in skeletal muscle, but the mechanism of angiogenesis in skeletal muscle remains unknown. In this study, we investigated the influence of differences in hypoxia exposure on angiogenesis in skeletal muscles at differing ages and metabolic characteristics at which the production of reactive oxygen species and nitric oxide may differ. Ten-week-old (young) and 20-month-old (old) mice were separated into control (N), continuous hypoxia (H), and intermittent hypoxia (IH) groups. The H group was exposed to 16% O_2_ hypoxia for 5 days and the IH group was exposed to 16% O_2_ hypoxia at one-hour intervals during the light period for 5 days. After completion of hypoxia exposure, the soleus and gastrocnemius muscles were immediately excised, and mRNA expression of angiogenesis- and satellite cell-related genes was investigated using real-time RT-PCR. In addition, muscle fiber type composition, muscle fiber area, number of satellite cells, and capillary density were measured immunohistochemically. In the young soleus muscle, the muscle fiber area was decreased in the H group, and mRNA expression of satellite cell activation-related MyoD, MHCe, and BDNF was significantly increased. On the other hand, in the old soleus muscle, nNOS and VEGF-A mRNA expression, and the capillary density were significantly increased in the H group. In the superficial portion of the gastrocnemius, mRNA expression of FGF2, an angiogenic factor secreted by satellite cells, was significantly increased in the young IH group. In addition, a positive correlation between VEGF-A mRNA expression and nNOS mRNA expression in the soleus muscle and eNOS mRNA expression in the superficial portion of the gastrocnemius was noted. These data demonstrated that age, hypoxia exposure method and muscle metabolic characteristics are related, which results in significant differences in angiogenesis.

## Introduction

A hypoxic environment [1] and exercise stimulation [2] are factors that increase reactive oxygen species (ROS), and hypoxic training combining these may markedly increase ROS [3]. Increases in ROS and reactive nitrogen species (RNS) may be necessary to acquire beneficial effects of exercise through hormesis effects of ROS and RNS on mitochondrial biosynthesis, angiogenesis and satellite cell (SC) activation [4, 5]. We previously demonstrated the beneficial effects of hypoxic training using thoroughbreds, but the capillary density was evaluated only in the gluteus medius muscle, which is a glycolytic metabolism-dominant fast twitch muscle [4]. In the previous study [6, 7] using hypoxia, muscle fiber type-specific adaptive changes were observed, suggesting that actions of ROS and RNS occur in a muscle fiber type-specific manner.

Continuous or intermittent exposure to hypoxia is known as a cause of increased ROS, and intermittent hypoxia markedly increases xanthine oxidase-mediated ROS production in reoxygenation when switching from hypoxia to normoxia [1, 8]. Although intermittent exposure to hypoxia has been combined with training to improve angiogenesis and endurance [9], it is unclear which method of hypoxic exposure is more effective.

Aging has been reported to increase the basal level of ROS but inhibit the exercise-induced additional increase in ROS [10]. In aged women, the VEGF-A mRNA response to exercise in the vastus lateralis muscle was lower than that in young women [11], but the VEGF-A mRNA response to acute hypoxia (FIO_2_ = 0.06, 2 h) and exercise in aged mice was similar to that in young mice [12]. Based on these findings, an increase in ROS in response to continuous and intermittent hypoxia exposure may be slow and the effects may weaken with age.

The objective of this study was to evaluate the effects of continuous or intermittent hypoxia in young (10 weeks old) and old mice (20 months old), especially on angiogenesis and SCs activation, employing immunohistochemical and real-time RT-PCR methods. Additionally, to examine muscle fiber type-specific adaptive responses to hypoxia, muscles (the soleus muscle and the superficial portion of gastrocnemius muscle) with different metabolic characteristics were analyzed.

## Materials and methods

### Animals and experimental design

All procedures were approved by the Animal Welfare and Ethics Committee of Yamaguchi University and followed the American Physiological Society’s Animal Care Guidelines.

**Fig 1:**
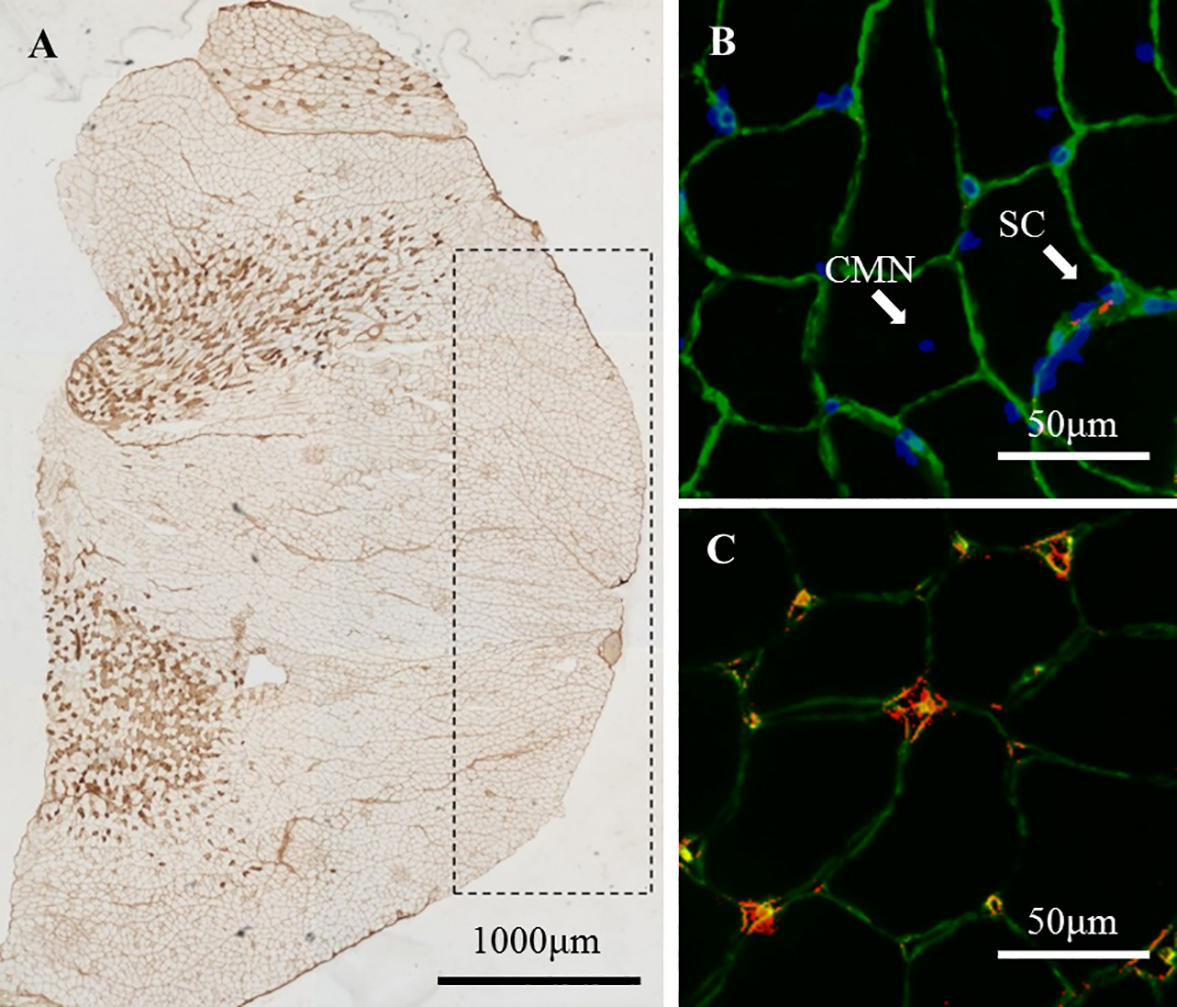
**Images for MHC-IIa (A), satellite cells (B), central myonucleus (B), and capillaries (C) in the gastrocnemius.** (A): Stained fibers represent MHC-IIa, and the area surrounded by the dashed line is the superficial portion of the gastrocnemius. (B) Image representing the basal lamina (green), myonucleus (blue), and satellite cells (red). White arrows indicate satellite cells (SCs) or central myonucleus (CMN). (C) Image representing co-localization of capillaries detected by laminin (green) and CD31 (red).

Sixteen young (10-week-old, body weight: 30.9±0.51 g) and seventeen old (20-month-old, body weight: 49.3±1.7 g) male mice (ICR-JCL strain) were supplied by Kyudo company (Tosu, Japan), and acclimated for at least 3 days before being used in experiments. Mice were randomly separated into normoxic control (N: FIO_2_ = 0.21), continuous hypoxia (H: FIO_2_ = 0.16), and intermittent hypoxia (IH: FIO_2_ = 0.21 and 0.16) groups. Normobaric hypoxia was achieved in a chamber (inside dimension: 28 x 35 x 24 cm) with an O_2_ controller (MC-8G-S; Iijima Electronics CO., Gamagori, Japan), and the CO_2_ concentration in the chamber was kept below 0.1% with a carbon dioxide monitor (COZY-1; JIKCO, Tokyo, Japan). The H group was housed in this chamber for 5 days, the IH group was subjected to repeated exposure of hypoxia and normoxia at 1-hour intervals for 12 hours, and spent 12 hours in a normoxic atmosphere for 5 days. All mice were housed in cages and maintained under artificial conditions at 23±2°C, with a constant humidity of 55±7% and 12- hour light / dark cycle. Solid food and water were provided ad libitum. After hypoxic exposure for 5 days, all animals were immediately anaesthetized with pentobarbital sodium (70 mg/kg, intraperitoneally), and then the left and right soleus and gastrocnemius muscles were removed. The entire soleus muscle and only the superficial portion of the gastrocnemius muscle were used for subsequent experiments (Fig 1A). All muscle samples were frozen by liquid nitrogen and stored at −80°C until analyzed.

### Immunohistochemical analysis

Fiber type population (%), cross-sectional area (CSA:μm^2^), SC number and capillary density were measured, as described previously [4]. Serial 10-μm cross sections of the muscle samples were obtained on a cryostat (CM510; Leica, Wetzlar, Germany) at −20°C. The sections were preincubated in 1% normal goat serum (EMD Millipore, Billerica, MA) in 0.1 M phosphate buffered saline (PBS; pH 7.6) at room temperature (RT) for 10 min. The primary monoclonal antibody was then applied: either (1) fast myosin (1: 2000; Sigma Aldrich, St. Louis, MO), which specifically reacts with the myosin heavy chain- (MHC-) IIa and IIx, or (2) SC-71 (1: 1000; Developmental Studies Hybridoma Bank, Iowa City, IA), which specifically reacts with MHC-IIa. The sections were incubated in these primary antibodies overnight at RT and incubated with a secondary antibody (goat anti-mouse IgG) conjugated with horseradish peroxidase (HRP, Bio-Rad, Hercules, CA, 1: 1,000) at RT for 3 hours. Diaminobenzidine tetrahydrochloride was used as a chromogen to localize HRP. Images of the stained muscle fibers were captured using a photomicroscope (BZ-X710, KEYENCE, Osaka, Japan). The fibers were classified as Type I, IIa or IIx/b fibers based on their immunohistochemical staining properties, and the population and CSA of each muscle fiber type were calculated from at least 200 muscle fibers.

Another cross-section of the muscle was fixed in 4% paraformaldehyde in 0.1 M PBS at RT for 10 min. These sections were preincubated in blocking solution containing 10% normal goat serum and 2% bovine serum albumin in PBS at RT for 30 min. Each section was incubated for 2 hours at RT in the primary antibodies mouse anti-paired box protein-7 (Pax7, Developmental Studies Hybridoma Bank; 1: 1,000) and rabbit anti-laminin (Sigma Aldrich, 1: 1,000) diluted in 2% bovine serum albumin/PBS. The sections were then incubated in the appropriate secondary antibodies: Cy3-conjugated AffiniPure goat anti-mouse IgG (Jackson ImmunoResearch, West Grove, USA; 1: 1,000) for Pax7 and Alexa Fluor 488 goat anti-rabbit IgG (Molecular Probes, Breda, Netherlands; 1: 1,000) for laminin. After incubation, the sections were stained with 4,6- diamidino-2-phenylindole (DAPI) diluted in PBS at RT for 5 min. Images for anti-Pax7, anti-laminin and DAPI were merged (BZ-X710, KEYENCE) and used for quantification of SCs (Fig 1B). SCs were identified as being positive for both DAPI and Pax7 at the periphery of each fiber beneath the basal lamina.

The capillaries surrounding clear basal lamina were counted, and presented as the capillary density (the number of capillaries per 1 mm^2^) and the capillary-to-fiber ratio in images for anti-laminin. The co-localization of these capillaries and CD31 was confirmed by another image of a 10-μm cross-section incubated with anti-laminin and anti-CD31 (1: 1000; Sigma Aldrich) instead of anti-Pax7, as described above (Fig 1C).

### RNA isolation and real-time RT-PCR

Relative quantification of mRNA expression in muscle samples (soleus and superficial portion of gastrocnemius) was performed by real-time RT-PCR, as described previously [4]. Real-time RT-PCR analysis of the gastrocnemius muscle was carried out by removing 10~20 mg from the region corresponding to the superficial portion (Fig 1A) in frozen muscle. Total RNA was isolated with TRIzol reagent (Molecular Probes, Breda, Netherlands) and then treated for 30 min at 37°C with TURBO DNase (Ambion, Austin, USA) to remove genomic DNA from the samples. First-strand cDNA was synthesized from the RNA (0.5 μg) with the Exscript RT reagent kit (Takara, Tokyo, Japan). Thereafter, the cDNA products were analyzed by real-time PCR using the SYBR Green PCR Master Mix protocol and StepOne Real-Time PCR System (Applied Biosystems Japan, Tokyo, Japan).

The amplification program included an initial denaturation step at 95°C for 10 min, 40 cycles of denaturation at 95°C for 30 sec and annealing/extension at 58°C for 1 min. The amount of glyceraldehyde-3-phosphate dehydrogenase (GAPDH) mRNA was estimated as an internal control. Each mRNA amount was normalized to GAPDH by subtracting the cycle threshold (Ct) value of GAPDH from the Ct value of the gene target [ΔCt (target)]. The relative expression of the target gene was calculated as the relative quantification value for the N group.

Primer sequences for RT-PCR are presented in Table 1. PCR primers for pax7, MyoD, myogenin, TNFα [13], Atrogin1, ATG5 [6] and BDNF [14] were made in reference to previous studies, whereas the others were designed by Primer Express software (Applied Biosystems Japan). The oligonucleotides were purchased from FASMAC (FASMAC, Kanagawa, Japan).

**Table 1:** Real-time RT-PCR primer sequences.

Gene	Forward sequence	Reverse sequence
GAPDH	CATGGCCTTCCGTGTTCCTA	GCGGCACGTCAGATCCA
Pax7	AAATCCGGGACCGGCTGCTGAA	AGACGGTTCCCTTTGTCGCCCA
MyoD	GGATGGTGTCCCTGGTTCTTCAC	CTATGTCCTTTCTTTGGGGCTGGA
myogenin	AACTACCTTCCTGTCCACCTTCA	GTCCCCAGTCCCTTTTCTTCCA
VEGF-A	AGTGGCTTACCCTTCCTCATCTT	CGGGTCCTGCCCCATT
FGF2	TGGTATGTGGCACTGAAACGA	TCCAGGTCCCGTTTTGGAT
BDNF	TAGCAAAAAGAGAATTGGCTG	TTTCAGGTCATGGATATGTCC
PGC1α	GGACAGTCTCCCCGTGGAT	TCCATCTGTCAGTGCATCAAATG
Neuronal NOS	GGTCTTCGGGTGTCGACAA	GAGTAGGCAGTGTACAGCTCTCTGA
Inducible NOS	GGATCTTCCCAGGCAACCA	CAATCCACAACTCGCTCCAA
Endothelial NOS	TTGTCTGCGGCGATGTCA	GAATTCTCTGCACGGTTTGCA
MHCe	GAGCAGCTGGCGCTGAA	TCTGATCCGTGTCTCCAGTTTCT
myostatin	ACCACGGAAACAATCATTACCAT	TGCCATCCGCTTGCATT
TNFα	ATGGCCTCCCTCTCATCAGT	CTTGGTGGTTTGCTACGACG
Atrogin1	ACCGGCTACTGTGGAAGAGA	CCTTCCAGGAGAGAATGTGG
ATG5	TTGAATATGAAGGCACACCCC	CTCTTGAAATGTACTGTGATGTTCCAA

GAPDH; glyceraldehyde-3-phosphate dehydrogenase, Pax7; paired box transcription factor-7, MyoD; myogenic determination factor, VEGF-A; vascular endothelial growth factor-A, FGF2; fibroblast growth factor 2, BDNF; brain-derived neurotrophic factor, PGC1α; proliferator-activated receptor gamma coactivator 1-alpha, NOS; nitric oxide synthase, MHCe; myosin heavy chain embryonic, TNFα; tumor necrosis factor alpha, ATG5; autophagy-related gene 5

### Statistics

All data are presented as the mean ± SE. Data obtained from the histochemical analysis and real-time RT-PCR were analyzed by two-way ANOVA (hypoxic method and age differences, S1 and S2 Tables) followed by the 𝑡-test with Bonferroni adjustment. Pearson's correlation coefficients between ratios of increases in mRNA expression were calculated based on total data for three experimental groups in each muscle from young (n = 16) and old (n = 17) mice. Significance was set at *P* < 0.05.

## Results

### Body and muscle weights

The muscle weights of young mice were lower in the H [soleus muscle (SOL): 13.3±0.3 g, gastrocnemius muscle (GA): 173.6±17.4 g] and IH [SOL: 13.4±0.6 g, GA: 173.7±5.2 g] groups than in the N group [SOL: 16.0±1.1 g, GA: 193.0±7.3 g], but not significantly. No change was observed in the muscle weights in all groups of old mice [(N group) SOL: 16.8±1.8 g, GA: 177.3±16.0 g, (H group) SOL: 17.0±0.6 g, GA: 178.2±3.9 g, (IH group) SOL: 15.9±1.1 g, GA: 197.6±16.1 g)].

### Muscle fiber properties

**Fig 2:**
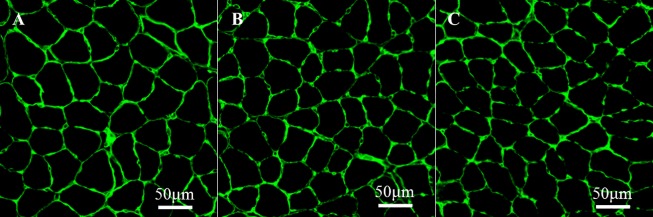
Images of muscle fibers in the young soleus muscle stained by laminin (green) from the N group (A), H group (B), and IH group (C).

The muscle fiber properties except for capillary densities are shown in Table 2. The fiber type populations for both muscles were similar among the all experimental groups for each age. For the soleus muscle in young mice, the CSA of type I (*P* = 0.022) and IIa (*P* = 0.006) fibers in the H group and type I (*P* = 0.002) fibers in the IH group was significantly lower than that in the N group (Fig 2). Except for the soleus muscle from young mice, the CSA of the muscle fiber was not significantly altered in response to hypoxia exposure. The myonuclear domain size of the soleus muscle in young mice was significantly lower because of the decrease in CSA of these muscles fibers [H: Type I (*P* = 0.001 in H vs. N), Type IIa (*P* = 0.003 in H vs. N), IH: Type I (*P* = 0.002 in IH vs. N)]. The ratios of SCs to muscle fibers for both muscles in young and old mice were slightly higher in the H and IH groups than in the N groups. The populations of fibers containing central nuclei were not significantly changed among experimental groups for each age, but significantly decreased from young to old mice in the N group (*P* = 0.02).

**Table 2:** Muscle fiber properties in each experimental group.

**SOL**	**Fiber type**	**Young**			**Old**		
		**N**	**H**	**IH**	**N**	**H**	**IH**
Population of muscle fiber type (%)	I	51.3±3.4	59.8±4.5	57.9±2.8	52.7±4	50.6±5	53.5±5.3
	IIa	48.7±3.4	40.2±4.5	42.1±2.8	47.3±4	49.4±5	46.5±5.3
Muscle fiber area (μm^2^)	I	2041±68	1563±115 †	1615±50 †	1915±74	1938±95	2215±240
	IIa	1734±76	1207±87 †	1419±87	1944±203	1966±80‡	1891±110‡
Myonuclear number/fiber	I	2.51±0.06	2.47±0.1	2.46±0.08	2.45±0.08	2.6±0.07	2.63±0.09
	IIa	2.27±0.07	2.09±0.12	2.13±0.1	2.36±0.1	2.44±0.05	2.19±0.08
Myonuclear domain size (μm^2^)	I	928±38	679±19 †	736±26 †	882±36	831±46‡	911±60
	IIa	854±32	637±27 †	754±34	925±69	880±33‡	953±62
Satellite cell number/100 fiber	I	3.3±1.1	4±1.3	6.1±1.3	2.2±1.1	3.4±1.1	5.0±1.7
	IIa	4±0.7	2.7±1.3	5.6±0.7	2.9±1.1	6±1.3	3.9±1.3
Fiber-containing central nucleus (%)	I	ND	ND	1.1±0.7	7.4±2.6	8.1±3.8	1.7±1.2
	IIa	ND	ND	0.6±0.6	7.9±3.2	5.3±3.9	2.2±1.1
**GA-S**	**Fiber type**	**Young**			**Old**		
		**N**	**H**	**IH**	**N**	**H**	**IH**
Population of muscle fiber type (%)	IIx/b	100±0	100±0	100±0	100±0	100±0	100±0
Muscle fiber area (μm^2^)	IIx/b	2518±38	2402±25	2723±111	2612±197	2513±151	2718±152
Myonuclear number/fiber	IIx/b	1.73±0.05	1.67±0.05	1.75±0.07	2.06±0.11	1.8±0.05	1.83±0.06
Myonuclear domain size (μm^2^)	IIx/b	1665±58	1658±36	1799±60	1469±153	1550±111	1671±112
Satellite cell number/100 fiber	IIx/b	5.3±1.3	6.0±1.3	7.8±3.4	5.0±0.8	6.0±1.3	5.0±0.8
Fiber-containing central nucleus (%)	IIx/b	1.3±0.8	3.3±1.1	2.8±1.6	15.6±3.6‡	8.7±2.5	11.1±3.3

Data are shown for properties of muscle fiber types and CSA in the soleus muscle (SOL) and superficial portion of the gastrocnemius (GA-S) in normoxic control (N), continuous hypoxia (H), and intermittent hypoxia (IH) groups of young and old mice. Values are the mean ± SE.

†: Significant difference from each N group (*P* < 0.05).

‡: Significant difference from young mice in each group (*P* < 0.05).

**Fig 3:**
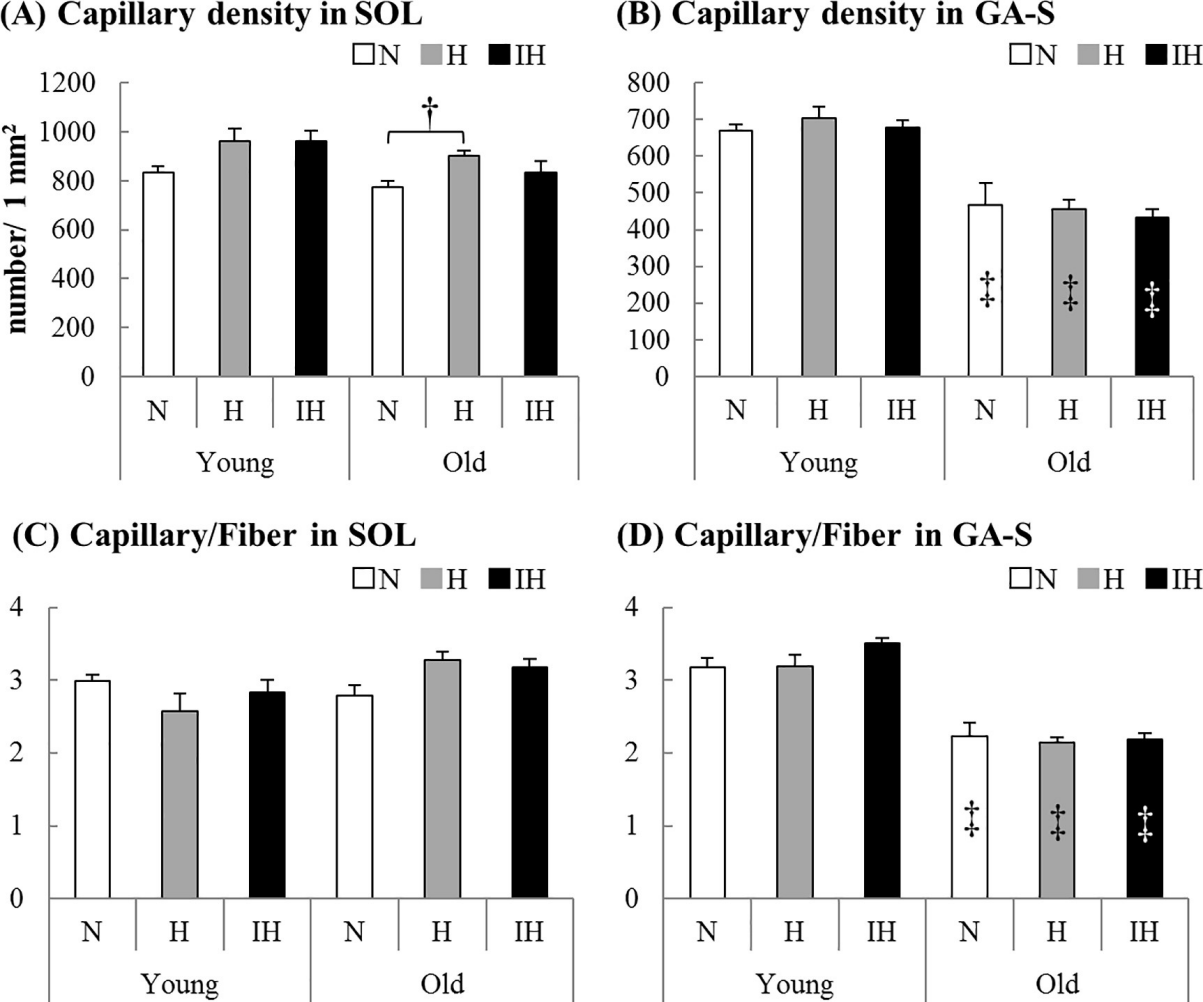
**The capillary density (number/ 1 mm**^**2**^**) and capillary-to-fiber ratio of the soleus (A, C) and superficial portion of the gastrocnemius (B, D) muscles in each experimental group of young and old mice (N: white bar, H: gray bar, IH: black bar).** Values are the mean ± SE. †: Significant difference from each N group (*P* < 0.05). ‡: Significant difference from young mice in each group (*P* < 0.05). The capillary density (A) was higher in the old soleus muscle of H groups than of N groups (*P* = 0.022). In old gastrocnemius muscles, capillary densities (B) and capillary-to-fiber ratios (D) in all groups were lower than those in young mice [capillary densities: N (*P* = 0.049), H (*P* = 0.001), IH (*P* = 0.001), capillary-to-fiber ratios: N (*P* = 0.001), H (*P* = 0.001), IH (*P* = 0.001)].

The capillary densities in the soleus muscle from old mice were significantly higher in the H group than in the N group (*P* = 0.022, Fig 3). Although there was no significant difference, the capillary density in the soleus muscle from young mice was ~15% higher after hypoxia exposure. Although the capillary to fiber ratio in the old soleus muscle in the H group was slightly higher than that in the N group, the ratios in all groups of both ages were not significant different from those in the N groups (Fig 3). In old gastrocnemius muscles, capillary densities and capillary to fiber ratios in all groups were lower than in those from young mice [capillary densities: N (*P* = 0.049), H (*P* = 0.001), IH (*P* = 0.001), capillary to fiber ratios: N (*P* = 0.001), H (*P* = 0.001), IH (*P* = 0.001)].

### Expression of mRNA

#### Soleus muscle

**Fig 4:**
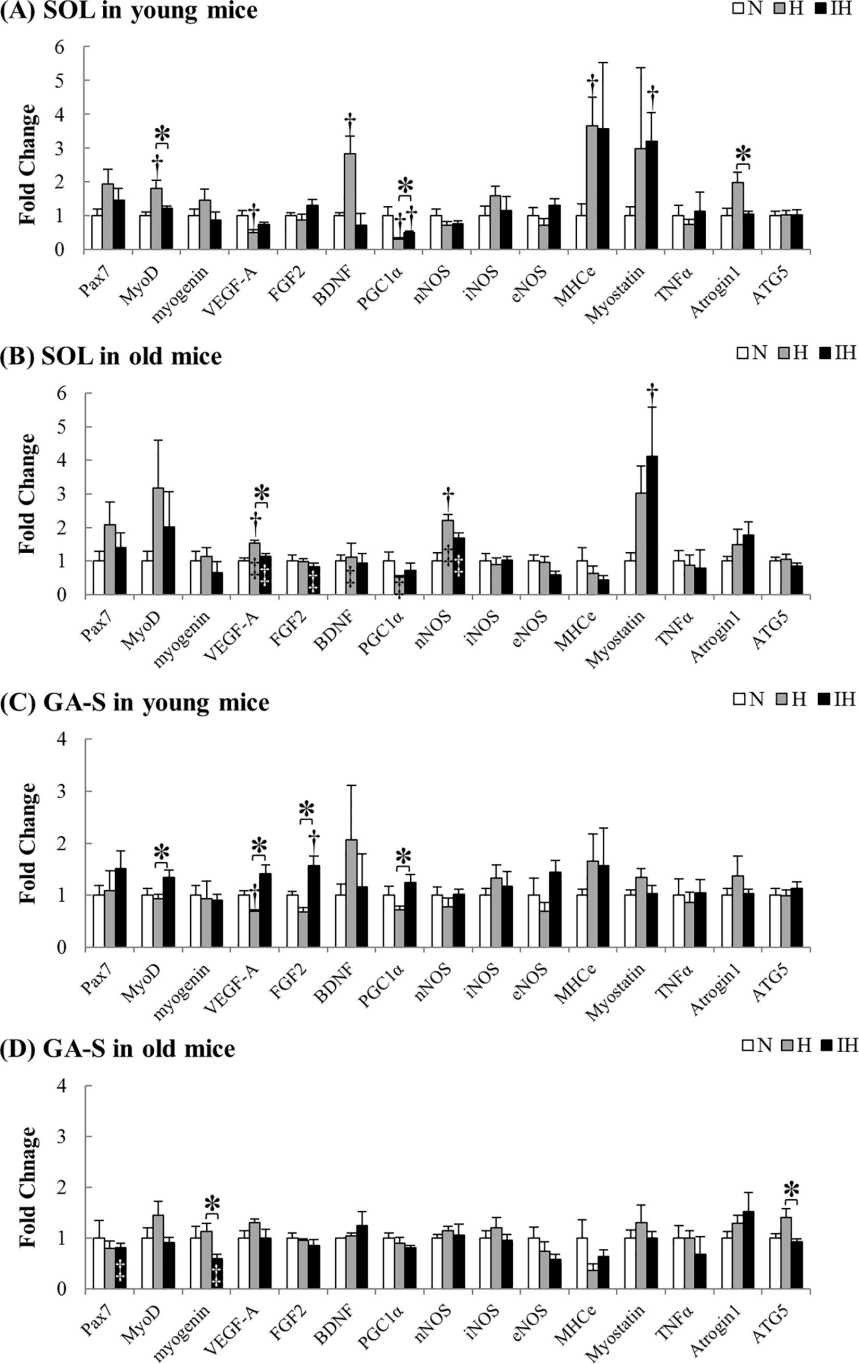
**mRNA expression in both hypoxic groups compared with each N group in the soleus (A, B) and superficial portion of the gastrocnemius (C, D) muscles from young and old mice.** Gray and black bars represent continuous hypoxia (H) and intermittent hypoxia (IH) groups, respectively. Values are the mean ± SE. †: Significant difference from the N groups for each gene (*P* < 0.05). *: Significant difference between H and IH groups for each gene (*P* < 0.05). ‡: Significant difference from young mice in each group (*P* < 0.05). (A) In the young soleus muscle, a significant increase was observed in the MyoD (*P* = 0.013 in H vs. N), BDNF (*P* = 0.002 in H vs. N), MHCe (P = 0.035 in H vs. N), Atrogin1 (*P* = 0.011 in H vs. IH), and myostatin (*P* = 0.020 in IH vs. N) mRNA expression. On the other hand, a significant decrease was observed in the VEGF-A (P = 0.044 in H vs. N) mRNA and PGC1α (*P* = 0.005 in H vs. N, *P* = 0.035 in IH vs. N) mRNA expression. (B) In the old soleus muscle, VEGF-A (*P* = 0.009) and nNOS (*P* = 0.035) mRNA expression was significantly increased. In the IH groups, myostatin mRNA expression was significantly higher than that in the N groups (*P* = 0.011). The mRNA expression in old mice was higher in VEGF-A (H: *P* = 0.001, IH: *P* = 0.001), nNOS (H: *P* = 0.001, IH: *P* = 0.001) and PGC1α (H: *P* = 0.001) than in the young mice, and was lower in BDNF (H: *P* = 0.039) and FGF2 (IH: *P* = 0.031) than in the young mice. (C) In the young gastrocnemius muscle, VEGF-A mRNA was significantly increased (*P* = 0.016 in H vs. N). FGF-2 mRNA expression was significantly higher than that in the N groups only in the gastrocnemius muscle of the IH groups (*P* = 0.028). In the IH groups, the expression of MyoD (*P* = 0.049), VEGF-A (*P* = 0.001), FGF-2 (*P* = 0.005), and PGC-1α (*P* = 0.014) mRNA was significantly higher than that in H groups. (D) The expression of myogenin (*P* = 0.024) and ATG5 (*P* = 0.028) mRNA was higher in the H groups than in the IH groups. The expression of pax7 (*P* = 0.022) and myogenin (*P* = 0.048) in the old IH groups was lower than that in the young IH groups.

The relative changes in mRNA expression for each muscle are shown as fold changes from the N group (Fig 4). In the young soleus muscle in the H group, the mRNA expression of MyoD (*P* = 0.013), BDNF (*P* = 0.002), and MHCe (*P* = 0.035) was significantly higher than that in the N group, and Atrogin1 (*P* = 0.011) mRNA expression was higher than that in the IH group (Fig 4A). On the other hand, the expression of VEGF-A (*P* = 0.044) mRNA in the H group and PGC1α mRNA in both hypoxic groups (*P* = 0.005, *P* = 0.035) was significantly lower than that in the N group. The myostatin mRNA expression in the young muscle was significantly higher in the IH than in the N groups (*P* = 0.020).

Unlike young mice, the VEGF-A (*P* = 0.009) mRNA expression in the old soleus muscle in the H group was significantly higher, and the nNOS (*P* = 0.035) mRNA expression was simultaneously increased (Fig 4B). Consistent with young mice, the myostatin mRNA expression in the old muscle in the IH group was significantly higher than that in the N group (*P* = 0.011). The mRNA expression of VEGF-A (H: *P* = 0.001, IH: *P* = 0.001) and nNOS (H: *P* = 0.001, IH: *P* = 0.001) in both hypoxic groups and the mRNA expression of PGC1α (*P* = 0.001) in the H groups were higher in the old muscle compared with young muscle in each group. On the other hand, the mRNA expression of BDNF (*P* = 0.039) in the H groups and the mRNA expression of FGF2 (*P* = 0.031) in the IH groups were lower in the old mice.

#### Gastrocnemius muscle

In the young gastrocnemius muscle in the H group, the expression of VEGF-A mRNA was significantly lower than that in the N group, similar to the soleus muscle (*P* = 0.016, Fig 4C). Only in the young muscle in the IH group, FGF-2 mRNA expression was significantly higher than that in the N group (*P* = 0.028). Furthermore, the expression of MyoD (*P* = 0.049), VEGF-A (*P* = 0.001), FGF-2 (*P* = 0.005), and PGC-1α (*P* = 0.014) mRNA in the young muscle in the IH group was significantly higher than that in the H group.

Although no significant differences were found in mRNA expression in the old gastrocnemius muscle in both hypoxic groups compared with the N group, the expression of myogenin (*P* = 0.024) and ATG5 (*P* = 0.028) mRNA was higher in the H group than in the IH groups (Fig 4D). Additionally, the expression of pax7 (*P* = 0.022) and myogenin (*P* = 0.048) in the old IH groups was lower than that in the young IH groups.

### Correlations between factors

Previous studies [15, 16] demonstrated that NO is involved in the activation of SCs, and activated SCs secrete angiogenic factors, including VEGF-A and FGF2. Thus, we analyzed correlations between the increased ratio of nNOS and eNOS mRNA expression, which are used to synthesize NO, and increased ratios of VEGF-A, FGF2, and MyoD mRNA expression, which are involved in angiogenesis and myogenesis in muscle (Table 3).

**Table 3:** Pearson's correlation coefficients between each factor.

		MyoD		VEGF-A		FGF2	
		SOL	GA-S	SOL	GA-S	SOL	GA-S
nNOS	Young	-0.16	0.16	0.67^✽^	0.25	-0.05	0.16
	Old	0.01	0.09	0.52^✽^	0.28	0.48	-0.1
eNOS	Young	0.24	0.50^✽^	0.14	0.59^✽^	0.5	0.35
	Old	0.32	0.31	-0.08	0.08	0.57^✽^	0.49^✽^

Pearson's correlation coefficients (R) between the ratio of the increase in nNOS, eNOS, and MyoD mRNA expression, and the ratio of the increase in MyoD, VEGF-A and FGF2 mRNA expression. Pearson's R was calculated based on total data for three experimental groups in each muscle from young and old mice.

*: Significant correlation between each factor (*P* < 0.05).

In the soleus muscle, there were significant correlations between the increased ratio of nNOS and VEGF-A mRNA expression in both age groups (young: *P* = 0.004, old: *P* = 0.033), and significant correlations between the increased ratio of eNOS and FGF2 mRNA expression in old mice (*P* = 0.017). On the other hand, in the gastrocnemius muscle, the increased ratio of eNOS mRNA expression was positively correlated with the increased ratio of MyoD (*P* = 0.049) and VEGF-A (*P* = 0.011) mRNA expression in young mice, and the increased ratio of FGF2 mRNA expression in old mice (*P* = 0.019).

## Discussion

### Muscle fiber properties

#### Soleus muscle

In the 5-day H and IH groups, the Type I and Type IIa fiber areas were significantly decreased in the young soleus muscle. On the other hand, no significant change was noted in the muscle fiber area in the old soleus muscle. In addition, in the young muscle, mRNA expression of a gene involved in proteolysis [17], Atrogin1, was significantly increased due to the reduction of the muscle fiber area in the H group compared with that in the IH group, and mRNA expression of myostatin, which is involved in the promotion of proteolysis and inhibition of muscle formation [18], was significantly increased in the IH group. Myostatin mRNA expression demonstrated a similar response in the old mice even though the muscle fiber area did not decrease. Myostatin mRNA expression has been reported to be increased through NF-kβ signaling in response to an increase in the ROS H_2_O_2_ [19], suggesting that this was associated with the significant increase observed only in the IH group.

In a previous study in which mice were continuously exposed to 8% O_2_ normobaric hypoxia for 3 weeks [6], the muscle fiber area did not change in the soleus muscle. Regarding the explanation for the different findings in the muscle fiber area between the previous and present studies, the intensity of hypoxia may have been related. In a study in which rats were exposed to 14–15% O_2_ intermittent hypoxia for 8 weeks, close to the condition in the present study, the muscle fiber area decreased with an increase in the capillary density without any change in the weight of the soleus muscle [20]. Relatively weak hypoxia may decrease the muscle fiber area in the soleus muscle independent of the muscle weight. The increase in the capillary density with no change in the muscle weight was considered to be due to apoptosis of existing muscle fibers and an increase in the number of muscle fibers in the previous study [20]. In the present study, mRNA expression of MyoD, MHCe, and BDNF were significantly increased in the young soleus muscle in the H group. Hypoxia has been reported to promote SC proliferation and differentiation in vitro, and MyoD increased as an important factor [21]. In addition, BDNF increases after exercise and is involved in muscle regeneration by new muscle fiber formation [22]. It has been reported that muscle-specific knockout of BDNF inhibited myogenin and MHCe expression in vitro, and increased the number of unfused (mononuclear) myocytes expressing MHC [23]. Additionally, a previous study examining the combination of SC depletion and overactivity in the plantaris muscle reported that the importance of the addition of myonuclei mediated by SCs for muscle hypertrophy was higher in young mice (8 weeks old) than adult mice (16 weeks old) [24]. If hypoxia stressed the transcriptional ability of the myonucleus to govern the fiber area, it may be reasonable that reduction of the myonuclear domain size and activation of SCs in response to hypoxia was observed only in young mice in this study. These results of the previous and present studies suggested that hypoxic stimulation promotes new muscle fiber formation or the addition of myonuclei in the soleus muscle of young animals. This SC activation may increase the capillary density without changing the muscle weight, as observed in the previous study.

#### Gastrocnemius muscle

No significant reduction of the muscle fiber area was noted in the superficial portion of the gastrocnemius in young or old mice. Similarly, no significant change was observed in mRNA expression of Atrogin1, ATG5, or myostatin by real-time RT-PCR, demonstrating that unlike the previous report [6] in which 8% O_2_ hypoxia induced significant glycolytic reduction of the muscle fiber area in the extensor digitorum longus muscle, the intensity of continuous and intermittent 16% O_2_ hypoxia was too low to cause glycolytic muscular atrophy. Furthermore, unlike the soleus muscle, MyoD and BDNF mRNA expression did not increase in the gastrocnemius in the young and old H groups, suggesting that together with changes in the muscle fiber area, the influence of hypoxia is weaker on the gastrocnemius than on the soleus muscle.

On the other hand, MyoD mRNA expression was significantly increased in the IH group compared with that in the young H group. This increase in MyoD mRNA expression was not accompanied by increases in BDNF or MHCe mRNA, compared with that in the N and H groups, suggesting that intermittent exposure to hypoxia activates SCs in the young gastrocnemius and that its mechanism is different from that in the soleus muscle in the H group. This increase in MyoD mRNA was absent in the old mice. We consider that the difference between the young and old mice can be explained by differences in the increase in the amount of ROS induced by intermittent exposure to hypoxia [10], SC activation ability [25], and reduction of NOS activity [26].

### Angiogenesis

#### Soleus muscle

A previous study [6] demonstrated that continuous exposure to 8% O_2_ hypoxia promotes angiogenesis in the soleus muscle in 12-week-old mice. In the present study, continuous exposure to 16% O_2_ hypoxia significantly increased the capillary density in the soleus muscle in 20-month-old mice. In addition, VEGF-A mRNA and nNOS mRNA were significantly increased, and a significant positive correlation was noted between these factors. Although this significant difference was observed in only H groups, the mRNA expression of VEGF-A and nNOS in both hypoxic groups was significantly higher in the old mice than in the young mice. nNOS has been reported to increase during exercise in humans and to be involved in angiogenesis [27], and VEGF-A mRNA and the number of capillaries were significantly decreased in nNOS-deficient mice [28]. In addition, an increase in the nNOS mRNA expression level under hypoxia has been reported [29]. The present study demonstrated that intermittent 16% O_2_ hypoxia induced increases in nNOS and VEGF-A mRNA expression in the soleus muscle in the old mice. Increases in both factors are considered to be involved in the significant increase in the capillary density observed in the old soleus muscle. As the increased capillary density was due to the decrease in muscle fiber area under hypoxia [6], the absence of increased nNOS or VEGF-A mRNA may have been related to the reduction of the muscle fiber area observed in the young soleus muscle. Regarding the significant reduction of VEGF-A mRNA expression, contradictory findings were noted in previous study: hypoxia (6% O_2_/ 2 h) [30] increased or decreased (12% O_2_/ 8 weeks) [31] mRNA expression. The difference due to the experimental conditions suggested that the basal level of VEGF-A mRNA expression increases in response to shorter or more intense hypoxia. In contrast, VEGF mRNA expression may not have been stimulated by long-term exposure (8 weeks) because capillaries sufficiently developed [31]. In the young soleus muscles in our study, the capillary density slightly increased due to the reduction of the muscle fiber area. The reduction of the muscle fiber area suggested that shortening of the diffusion distance weakened angiogenic stimulation, similar to the conditions in which capillaries sufficiently developed in response to long-term exposure.

Intermittent hypoxia markedly increases xanthine oxidase-mediated ROS production in reoxygenation [8]. ROS is associated with several impairments and is also involved in the beneficial effects of exercise [5]. Indeed, intermittent exposure to moderate hypoxia has been suggested to be beneficial for blood flow and blood pressure [32]. In the IH group, ROS did not significantly change the number of capillaries in the soleus muscle in young and old mice. In addition, in the old mice, VEGF-A and nNOS mRNA expression was not influenced, unlike in the H group. This may have been due to the short exposure to hypoxia compared with the H group.

#### Gastrocnemius muscle

The capillary density and capillary-to-fiber ratio in old mice were lower than those in young mice. These results are consistent with previous human studies that reported that these parameters simultaneously reduced in vastus lateralis muscle with aging [33].

In the superficial portion of the gastrocnemius continuously exposed to hypoxia, no correlation was noted between nNOS and VEGF-A mRNA expression in young and old mice, unlike in the soleus muscle, and the number of capillaries did not increase. In the previous study, exposure to more intense hypoxia (8% O_2_) caused atrophy of the extensor digitorum longus muscle [6], whereas exposure to 16% O_2_ did not significantly change the muscle fiber area in the present study. This result also suggested that the diffusion distance was not shortened by the reduction of the muscle fiber area, unlike that in the young soleus muscle.

NO, which is involved in vascular dilatation, is synthesized by constitutive NOS (eNOS and nNOS) [34]. In NO synthesis in a hypoxic environment, eNOS activity decreases, whereas the influence of nNOS increases [34]. The nNOS was more strongly expressed in Type IIb fibers than in other fiber types in a previous study [35]. The nNOS expression level is considered to be higher in the gastrocnemius than in the soleus muscle suggested that an increase in blood flow helped alleviate hypoxia. On the other hand, in the previous study employing intense hypoxia (8% O_2_) [6], reduction of the muscle fiber area in the extensor digitorum longus muscle was observed, suggesting that the upregulation of nNOS [29] or NO-induced increase in blood flow may have reached the upper limit under hypoxia.

An increase in blood flow upregulates eNOS mRNA expression by increasing shear stress, which is involved in angiogenesis [36]. Indeed, in previous study using the mouse extensor digitorum longus muscle, both overactivity and an increase in blood flow caused by prazosin (vasodilator: the α1-adrenergic receptor antagonist) administration induced angiogenesis, but angiogenesis did not occur only in the prazosin treatment group of eNOS-deficient mice [37]. It has also been reported that neither overactivity nor increased blood flow was inhibited in nNOS-deficient mice, clarifying that the increase in blood flow induces angiogenesis in association with eNOS, but not in association with nNOS. In the previous study using a similar experimental system, inhibition of VEGF-A abolished the angiogenic actions of both overactivity and prazosin administration, suggesting that VEGF-A is a mediator of eNOS induced by an increase in blood flow and angiogenesis [38]. In the present study, VEGF-A and eNOS mRNA expression was higher in the IH group than in the H group, and a significant positive correlation was noted between the increases in eNOS mRNA and VEGF-A mRNA only in the young gastrocnemius. Vascular dilatation occurs during exercise under hypoxia compared with that under normoxia, but this vascular dilatation was found to be weak in the elderly compared with that in the young, and NOS inhibitor-induced reduction of vascular dilatation was not observed in the old [26]. These findings may explain the absence of a positive correlation between the 2 factors in the old gastrocnemius in the present study.

It is possible that downregulation of eNOS expression under hypoxia [34] is related to significant differences in the eNOS and VEGF-A mRNA expression levels in the young gastrocnemius between the H and IH groups. In a study involving humans, high-intensity interval training markedly increased the capillary density and eNOS protein amount compared with those induced by endurance training [39], suggesting that interval training is more effective than continuous exercise to increase eNOS.

In the young gastrocnemius, VEGF-A mRNA was significantly increased and MyoD mRNA was increased in the IH group compared with those in the H group, suggesting that SCs are involved in eNOS-VEGF-A-induced angiogenesis. Indeed, SCs are activated by NO [15], and these cells have been reported to release angiogenic factors such as VEGF-A and FGF2 [16]. In the present study, FGF2 mRNA was significantly increased in the young superficial portion of the gastrocnemius in the IH group. In addition, eNOS mRNA expression was significantly correlated with MyoD and VEGF-A mRNA expression in the young gastrocnemius, suggesting that SCs mediate increases in NO-induced VEGF-A mRNA in the young gastrocnemius.

## Conclusions

In young mice, exposure to continuous hypoxia decreased the muscle fiber area and concurrently increased the expression of satellite cell-related genes in the soleus muscle, but not in the gastrocnemius. Furthermore, in old mice, exposure to continuous hypoxia increased the mRNA expression of nNOS and VEGF-A, and the capillary density in the soleus muscle, but not in the gastrocnemius. We concluded that differences in the age of the mice, hypoxia exposure method, and muscular metabolic characteristics led to significant differences in angiogenesis in skeletal muscle.

## Supporting information

S1 Table Results of ANOVA in data of Table 2 and Fig 3. (PDF)

S2 Table Results of ANOVA in data of Fig 4. (PDF)
