# Supplementary material for: Influence of hypoxic stimulation on angiogenesis and satellite cells in mouse skeletal muscle
Source: PLoS One. 2018 Nov 8;13(11):e0207040. doi: 10.1371/journal.pone.0207040 (PMC6224099; doi:10.1371/journal.pone.0207040)
Supplement: S1 Table — (PDF) [file pone.0207040.s001.pdf]

S1 Table. Results of ANOVA in data of Table 2 and Figure 3

**SOL**	Fiber type	Hypoxia effect	Aging effect	Interaction	**GA-S**	Fiber type	Hypoxia effect	Aging effect	Interaction
Population of muscle fiber type (%)	I	N.S.	N.S.	N.S.	Population of muscle fiber type (%)	IIx/b	N.S.	N.S.	N.S.
	IIa	N.S.	N.S.	N.S.					
Muscle fiber area (μm^2)	I	$P < 0.05$	N.S.	N.S.	Muscle fiber area (μm^2)	IIx/b	N.S.	N.S.	N.S.
	IIa	$P < 0.05$	$P < 0.05$	N.S.					
Myonuclear number/fiber	I	N.S.	N.S.	N.S.	Myonuclear number/fiber	IIx/b	N.S.	N.S.	N.S.
	IIa	N.S.	N.S.	N.S.					
Myonuclear domain size (μm^2)	I	$P < 0.05$	$P < 0.05$	N.S.	Myonuclear domain size (μm^2)	IIx/b	N.S.	N.S.	N.S.
	IIa	$P < 0.05$	$P < 0.05$	N.S.					
Satellite cell number/100 fiber	I	N.S.	N.S.	N.S.	Satellite cell number/100 fiber	IIx/b	N.S.	N.S.	N.S.
	IIa	N.S.	N.S.	N.S.					
Fiber-containing central nucleus (%)	I	N.S.	N.S.	N.S.	Fiber-containing central nucleus (%)	IIx/b	N.S.	$P < 0.05$	N.S.
	IIa	N.S.	N.S.	N.S.					
Capillary density	I+IIa	$P < 0.05$	N.S.	N.S.	Capillary density	IIx/b	N.S.	$P < 0.05$	N.S.
Capillary/fiber	I+IIa	N.S.	$P < 0.05$	$P < 0.05$	Capillary/Fiber	IIx/b	N.S.	$P < 0.05$	N.S.
