# Supplementary material for: Influence of hypoxic stimulation on angiogenesis and satellite cells in mouse skeletal muscle
Source: PLoS One. 2018 Nov 8;13(11):e0207040. doi: 10.1371/journal.pone.0207040 (PMC6224099; doi:10.1371/journal.pone.0207040)
Supplement: S2 Table — (PDF) [file pone.0207040.s002.pdf]

S2 Table. Results of ANOVA in data of Figure 4

**SOL**	Hypoxia effect	Aging effect	Interaction	**GA-S**	Hypoxia effect	Aging effect	Interaction
Pax7	N.S.	N.S.	N.S.	Pax7	N.S.	$P < 0.05$	N.S.
MyoD	$P < 0.05$	N.S.	N.S.	MyoD	$P < 0.05$	N.S.	N.S.
myogenin	N.S.	N.S.	N.S.	myogenin	$P < 0.05$	$P < 0.05$	N.S.
VEGF-A	$P < 0.05$	$P < 0.05$	N.S.	VEGF-A	$P < 0.05$	N.S.	N.S.
FGF2	N.S.	$P < 0.05$	N.S.	FGF2	$P < 0.05$	N.S.	$P < 0.05$
BDNF	$P < 0.05$	$P < 0.05$	N.S.	BDNF	$P < 0.05$	$P < 0.05$	N.S.
PGC1α	$P < 0.05$	$P < 0.05$	N.S.	PGC1α	$P < 0.05$	N.S.	N.S.
nNOS	$P < 0.05$	$P < 0.05$	N.S.	nNOS	N.S.	N.S.	N.S.
iNOS	N.S.	N.S.	N.S.	iNOS	N.S.	N.S.	N.S.
eNOS	N.S.	$P < 0.05$	$P < 0.05$	eNOS	N.S.	N.S.	N.S.
MHCe	$P < 0.05$	N.S.	$P < 0.05$	MHCe	N.S.	N.S.	N.S.
Myostatin	$P < 0.05$	N.S.	N.S.	Myostatin	N.S.	N.S.	N.S.
TNFα	N.S.	N.S.	N.S.	TNFα	N.S.	N.S.	N.S.
Atrogin1	$P < 0.05$	N.S.	N.S.	Atrogin1	N.S.	N.S.	N.S.
ATG5	N.S.	N.S.	N.S.	ATG5	$P < 0.05$	N.S.	N.S.
